# Effect of the Antimicrobial Agents Peppermint Essential Oil and Silver Nanoparticles on Bone Cement Properties

**DOI:** 10.3390/biomimetics7030137

**Published:** 2022-09-17

**Authors:** Alina Robu, Aurora Antoniac, Robert Ciocoiu, Elena Grosu, Julietta V. Rau, Marco Fosca, Ivan I. Krasnyuk, Gratiela Gradisteanu Pircalabioru, Veronica Manescu (Paltanea), Iulian Antoniac, Sebastian Gradinaru

**Affiliations:** 1 Faculty of Material Science and Engineering, University Politehnica of Bucharest, 313 Splaiul Independentei Street, District 6, 060042 Bucharest, Romania; 2 Istituto di Struttura della Materia, Consiglio Nazionale delle Ricerche (ISM-CNR), Via del Fosso del Cavaliere, 100, 00133 Rome, Italy; 3 Institute of Pharmacy, Department of Analytical, Physical and Colloid Chemistry, I.M. Sechenov First Moscow State Medical University, Trubetskaya 8, Build. 2, 119991 Moscow, Russia; 4 Research Institute of the University of Bucharest, University of Bucharest, 90 Soseaua, Panduri, 050663 Bucharest, Romania; 5 Academy of Romanian Scientists, 54 Splaiul Independentei Street, District 5, 050094 Bucharest, Romania; 6 Faculty of Electrical Engineering, University Politehnica of Bucharest, 313 Splaiul Independentei, District 6, 060042 Bucharest, Romania; 7 Faculty of General Medicine, University of Medicine and Pharmacy Carol Davila Bucharest, 050474 Bucharest, Romania

**Keywords:** bone cements, antimicrobial agents, mechanical properties, TGA, biocompatibility

## Abstract

The main problems directly linked with the use of PMMA bone cements in orthopedic surgery are the improper mechanical bond between cement and bone and the absence of antimicrobial properties. Recently, more research has been devoted to new bone cement with antimicrobial properties using mainly antibiotics or other innovative materials with antimicrobial properties. In this paper, we developed modified PMMA bone cement with antimicrobial properties proposing some experimental antimicrobial agents consisting of silver nanoparticles incorporated in ceramic glass and hydroxyapatite impregnated with peppermint oil. The impact of the addition of antimicrobial agents on the structure, mechanical properties, and biocompatibility of new PMMA bone cements was quantified. It has been shown that the addition of antimicrobial agents improves the flexural strength of the traditional PMMA bone cement, while the yield strength values show a decrease, most likely because this agent acts as a discontinuity inside the material rather than as a reinforcing agent. In the case of all samples, the addition of antimicrobial agents had no significant influence on the thermal stability. The new PMMA bone cement showed good biocompatibility and the possibility of osteoblast proliferation (MTT test) along with a low level of cytotoxicity (LDH test).

## 1. Introduction

Bone cement is one of the most important biomaterials in hip and knee arthroplasty, and its quality has a great influence on the prosthesis survival rate [1,2]. According to the Canadian Institute for Health Information (CIHI), 84,770 secondary total knee arthroplasty surgeries were done between 2012 and 2020, and their main causes were due to infections (38.4%), prosthesis instability (22.7%), and aseptic loosening (16.5%). It was estimated that about 48,000 hip and knee surgeries were not performed in the last two years due to the pandemic situation, and doctors performed 6.6% of all joint replacements as day surgeries [3,4]. Poly (methyl methacrylate) (PMMA) bone cement is usually used to fix knee or hip prostheses. In some cases, it can act as a temporary spacer in the revision of second-stage surgeries. PMMA bone cement and other biomaterials can be loaded with different chemical substances or drugs to fight against infections that can appear after surgical intervention in different surgical specializations [5,6,7,8,9,10,11].

It is well known that PMMA cement is an inert material, which does not stimulate the osteointegration of the prosthesis, and its surface can be contaminated with different bacterial or microbial species inside the human body. Bone cement is loaded with antibiotics to prevent the infections associated with the implants. However, there are still debates regarding the adequate concentration and quantity of the drugs in order not to have harmful effects on organs [12]. Also, it is essential to know precisely how the active substances are delivered. Some literature references reported that adding drugs to PMMA bone cement affects the mechanical properties [13,14].

Usually, the bone cement is prepared in the surgery room by mixing a liquid component consisting of the monomer (methyl methacrylate) (MMA), to which one adds N, N-dimethyl-p-toluidine that sustains the polymerization process, hydroquinone as a stabilizer, powders comprised from PMMA particles, benzoyl peroxide, zirconia, and barium sulfate as radio-opacifier substances. The preparation process includes four stages as follows: (1) mixing phase, (2) waiting phase, (3) working phase, and (4) hardening or setting phase [12]. The polymerization reaction is exotherm, and the temperature at which the process occurs is influenced by different factors, such as the thickness of the cement layer and endosteal or periosteal blood circulation. Classical PMMA cement exhibits high biocompatibility, but unfortunately, it is characterized by low mechanical properties such as low compression, fatigue, and breaking resistance. The main problems linked with PMMA use are the improper mechanical bond between cement and bone and the absence of antibacterial properties [15,16].

Recently, much research has been devoted to improving bone cement’s antibacterial properties [17,18]. The most used substances are gentamicin [19], vancomycin [20], tobramycin [21], clindamycin [22], cephalosporine [23], ciproflaxcin [24], and tetracycline [25,26]. In this way, the PMMA cement delivers a high quantity of drugs at the infection site without adverse effects on other human body organs. In order to be incorporated into bone cement, an antibiotic must be available in powder form, have bactericidal properties at low concentrations, exhibit thermal stability, have a low influence on the mechanical properties of the cement, and have a low to no risk of allergy [27]. Glycopeptides and aminoglycosides are antibiotics that are very well suited to this type of use. Unfortunately, bacteria have developed a genetic and biochemical mechanism to fight against antibiotics, so as a consequence, other innovative solutions must be searched out. Firstly, an antibiogram is necessary, but in some cases, it cannot be used because drugs mixed with cement lose their antibacterial properties and can be inactivated. A low quantity of antibiotics might limit the proper application of bone cement [28,29].

Another solution found in the literature consists of incorporating gold or silver nanoparticles in PMMA cement. Nanomaterials exhibit specific physical–chemical properties such as a high surface area to mass ratio, minimal diffusion restrictions, and high activity. The addition of nanoparticles could improve the cement’s mechanical and antibacterial properties [30]. Zurek et al. [30] and Wekwejt et al. [31] studied the effect of nanosilver or nanocopper on the biomechanical properties of the cement. Microhardness, curing time, wettability, and mechanical tests were carried out. The flexural modulus for cement combined with nanosilver was about 2.9 GPa, and the flexural strength was found to be 69 MPa. These values are close to those reported for pure cement, with flexural modulus values between 2.8 GPa and 3.5 GPa. It was concluded that the concentration of silver nanoparticles is critical in cement toxicity reduction. Prokopovich et al. [32] developed silver–tiopronin nanoparticles with particle diameters between 5 nm and 11 nm and mixed them with PMMA cement in different concentrations between 0.1% *w*/*w* and 1% *w*/*w*. The presence of silver nanoparticles did not greatly influence the material’s resistance to compression. Additionally, they found that in the case of small-diameter particles, no bactericidal effects were in evidence. For the bone cement loaded with large-diameter nanosilver, an important reduction of Methicillin-Resistant *Staphylococcus aureus* (MRSA) contamination was noticed. Ag-nanoparticles functionalized with polyvinylpyrrolidone incorporated in commercial bone cement proved to have antibacterial effects on *Staphylococcus aureus* and *Staphylococcus epidermis* biofilms but exhibited no effect on planktonic bacteria [33]. Other solutions for increasing the antibacterial effect of PMMA cement consist of gold nanoparticle inclusion, which showed in [34] high antibacterial activity against MRSA and *Pseudomonas aeruginosa* biofilms. Bone cement with increased biocompatibility and bioactivity, such as that developed by Phakatkar et al. [35], through the inclusion of magnesium phosphates, has an important antibacterial effect on different types of bacteria. Tijana et al. [36] investigated the mechanical properties of PMMA/gold nanoparticles (AuNPs) composite with enhanced antibacterial properties. They concluded that the elastic modulus and flexural strength decreased for all the materials that contain AuNPs. The Vickers hardness value is directly proportional to increases in the gold nanoparticles’ percent. A value of 21.45 HV was reported for 0.74 wt.% of AuNPs.

Essential oils produced from aromatic plants, due to the secondary metabolism route, proved to influence fungi, bacteria, and yeasts. Sartoratto et al. [37] investigated the antimicrobial activity of different essential oils extracted from *Mentha piperita*, *Thymus vulgaris*, *Origanum vulgare*, *Aloysia triphylla*, and *Ocimum gratissimum,* among others. Antibacterial properties were tested against *Pseudomonas aeruginosa*, *Rhodococcus equi*, *Salmonella choleraesuis*, *Micrococcus luteus*, *Staphylococcus aureus*, *Staphylococcus epidermis,* etc. Different results were obtained, and it was concluded that all the investigated essential oils had antibacterial properties. However, they are adequate only in the case of a limited number of pathogens. Accurate and detailed investigations must be carried out in order to understand which microorganisms can be eliminated using each type of essential oil. One of the most investigated essential oils is peppermint. It is well known that this oil exhibits antibacterial activity due to limonene, L-menthol, methyl acetate, and menthone [1]. Unalan et al. [38] investigated the physical and antibacterial properties of peppermint essential oil-loaded poly (ε-caprolactone) fibers produced through electrospinning dedicated to wound healing. The antimicrobial activity was tested against Gram-positive (*Staphylococcus aureus*) and Gram-negative (*Escherichia coli*) bacteria, and it proved to be a promising candidate for wound healing. Thosar et al. [39] tested the antimicrobial efficacy of five essential oils such as thyme oil, lavender oil, peppermint oil, and eugenol oil, against the following strains, *Staphylococcus aureus*, *Enterococcus faecalis*, *Escherichia coli*, and *Candida albicans*. It was noticed that the most critical effects against oral bacteria were exhibited by peppermint oil, tea tree oil, and thyme oil, apart from the traditional use of eugenol. Kasiri et Fathi [40] developed cellulose nanocrystals, which were extracted from pistachio shells, in which they encapsulated peppermint oil. Their final conclusion was that diffusion transport is the main mechanism that governs the release of peppermint oil. Robu et al. [1] tested a modified PMMA bone cement based on hydroxyapatite impregnated with peppermint oil. It proved crucial antibacterial properties against Gram-positive and Gram-negative bacteria. To analyze the biocompatibility of the novel bone cement, an MTT assay was applied in the case of the MG-63 human cell line, proving that the modified PMMA cement does not have toxic effects. Peppermint oil-modified cement is a promising material in orthopedy and can be introduced in the near future in common clinical practice.

In this paper, we develop innovative modified PMMA-based bone cement with antibacterial properties. The proposed solutions consist of silver nanoparticles incorporated in ceramic glass and peppermint oil included in hydroxyapatite use. The impact of the addition of antimicrobial agents on the structure, mechanical properties, and biocompatibility of elaborated bone cements was quantified. The contact area of the acrylic cement with the bone is intensely mechanically stressed, so determining the compressive strength is very important [41,42]. In hip joint prostheses, the cement mantle is a compression wedge between the bone and the femoral stem, acting as a shock absorber between the implant and the bone [43,44,45]. The results obtained for this innovative modified PMMA-based bone cement will be compared with those obtained in the case of commercial polymethylmethacrylate bone cement and polymethylmethacrylate bone cement loaded with gentamicin, a well-known antimicrobial agent.

## 2. Materials and Methods

The experimental samples were obtained using a polymethylmethacrylate cement available on the market. The powder phase was composed of poly(methyl methacrylate) (PMMA), benzoyl peroxide (BPO), and the radiopaque agent, barium sulfate (BaSO_4_). In contrast, the organic phase consisted of methyl methacrylate (MMA) and butyl methacrylate (BMA) monomers, the activator of polymerization (*N*,*N*-Dimethyl-p-toluidine, DmpT), and hydroquinone (HQ). As antimicrobial agents, we used silver nanoparticles incorporated in a ceramic glass matrix (as one commercial product from SANITIZED AG, Burgdorf, Switzerland), peppermint essential oil (from Hofigal Export Import SA, Bucharest, Romania) incorporated in hydroxyapatite (HAp, >95% purity, Plasma Biotal Limited, Tideswell, UK), and gentamicin (KRKA D.D., Novo Mesto, Slovenia) according to Table 1.

The silver nanoparticles were incorporated into the bone cement composition in a proportion of 2% (AM1 sample) and 4% (AM2 sample), respectively. Sample HUM contains 5% peppermint essential oil incorporated in hydroxyapatite, and sample GH contains 5% gentamicin. The commercial polymethylmethacrylate cement (R sample) was used as a reference.

### 2.1. Energy Dispersive X-ray Diffraction (EDXRD)

The EDXRD diffractometer is home-assembled equipment that is used in the Energy Dispersive X-ray Diffraction technique. It consists of white X-ray radiation produced by a commercial W-anode X-ray tube (up to 50 keV) and a solid-state detector, in our case, an EG&G high purity Germanium photodiode, with an energy resolution of approximately 1.5–2.0% in the 20–50 keV energy range with the capability to perform an energy scan. The ADCAM hardware connects the detector to a PC, and Maestro software processes the signal and completes the required analog to digital conversions. The energy scan is carried out electronically, and diffraction patterns represent the diffracted intensity (n° of incident X-photons) as a function of the scattering parameter q (q = aE sinϑ), where q is the normalized momentum transfer magnitude, a is a constant, E is the incident X-ray beam energy, and 2ϑ is the scattering angle). As preliminary experiments to determine the optimal experimental conditions, ex situ diffraction patterns were recorded for powder samples at different scattering angles. All the measurements were performed with a white primary beam with energy ranging from 0 to 50 KeV and with a current intensity of 30 mA. Based on previous experience, we found that the best scattering angle is around 2θ = 10°. Peaks at around 2θ = 10° are the fluorescence signals of the W anode X-ray tube used as a radiation source of the diffraction apparatus. Those peaks are registered at around 8.5 KeV (W Lα) and 9.5 KeV (W Lβ), and upon conversion, they are reported (in this specific case) at around 2θ = 10°. As for the Ba fluorescence peaks, they are considered not relevant for the diffraction analysis and therefore are not discussed. The difference in relative intensities can be attributed to different scattering angles acquisition and normalization processes of the EDXRD patterns.

### 2.2. Raman Spectroscopy

Raman spectroscopy was conducted through the Jobin-Yvon-Horiba micro-Raman system (LabRAM ARAMIS) equipped with a solid-state laser and a diode laser with wavelengths of 532 nm and 785 nm, respectively, as excitation sources. The Horiba micro-spectrometer is coupled with a confocal microscope that allows the spatial resolution of the sample through the detector pinhole aperture. The advantage of the confocal microscope lies in its ability to remove signals from different layers or out-of-focus sample volumes. The spectrometer is equipped with a 2400 lines/mm diffraction grating connected to a CCD camera. Using an ultra-long working distance (10×) objective, the laser light reached the sample surface with normal incidence. Backscattering geometry was utilized to gather the scattered radiation. The fluorescent background from the Raman spectra was removed using a polynomial fit. Simultaneously, deconvolution of Raman patterns was performed by nonlinear least-squares fitting of the Raman peaks to a combination of Lorentzian and Gaussian line forms, yielding the peak height, width, position, and integrated intensity of each Raman band.

### 2.3. Mechanical Characterization

The new formulations and conventional (used as reference) ones for bone cement were tested to determine their mechanical characteristics in compression and flexure, loading types that are more likely to occur in service.

For each formulation, five test specimens were prepared by forming in a Teflon mold, extracted post-polymerization, and left to cure for 48 h in laboratory conditions. For compression tests, 20 mm in diameter and 40 mm in height were used for cylindrical specimens. For flexural tests, prismatic specimens 90 mm × 15 mm × 7 mm were prepared.

Both tests were performed on a universal testing machine, Walter + Bai LFV300 (Walter+bai AG, Löningen, Switzerland) equipped with steel compression platens and a three-point bending device comprising three steel cylinders with a diameter of 25 mm (two rollers for supporting the specimen and the third the loading nose) for the flexure test.

The compression tests were performed according to ASTM D695-15 “Standard Test Method for Compressive Properties of Rigid Plastics” and the flexure tests according to ASTM D790-15 “Standard Test Method for Flexural Properties of Unreinforced and Reinforced Plastics and Electrical Insulating Materials” specifications.

### 2.4. Thermogravimetric Analyses

Thermogravimetric analyses were performed using a TA Instruments SDT Q600 system. TGA data were recorded over the temperature range of 10–600 °C with a constant heating rate of 10 °C per minute in a nitrogen working atmosphere.

### 2.5. Biocompatibility Properties

G292 osteoblasts were cultured in Dulbecco’s Modified Eagle Medium (DMEM, Sigma-Aldrich, St. Louis, MO, USA). The medium was supplemented with 1% penicillin/streptomycin solution (Sigma-Aldrich) and 10% Fetal Bovine Serum (FBS, Sigma-Aldrich). Cells were cultured for 24 h at 37 °C in a humidified atmosphere, then washed with Phosphate Buffered Solution (Sigma-Aldrich). As the protocol of cell counting (using a hemocytometer) requires, the cells were harvested (using trypsin-EDTA from Sigma-Aldrich) and counted using Trypan Blue from Sigma-Aldrich and a hemocytometer. The samples (R, AM1, AM2, HUM, GH) were co-cultured with the cells (a seeding density of 5 × 10^5^ cells/well) for 24 h (37 °C, 95% humidity, 5% CO_2_). Cell viability and proliferation in the presence of the samples were estimated by MTT assay (using 3-(4,5-dimethylthiazol-2-yl)-2,5-diphenyltetrazolium bromide). Cells were incubated for 4 h with MTT reagent (Vybrant^®^ MTT Cell Proliferation Assay Kit, Invitrogen, Carlsbad, CA, USA, cat no. V-13154) at 37 °C, 95% humidity with 5% CO_2_. After incubation, formazan crystals were solubilized with dimethyl sulfoxide (DMSO) for 10 min at room temperature. Absorbance was measured using Mulsiskan FC (Thermo Scientific) at λ = 540 nm.

The cytotoxic potential of all investigated bone cement was evaluated by LDH assays. Lactate dehydrogenase (LDH) is an oxidoreductase present in most organisms. Cells that have lost their membrane integrity are released into the culture medium, the cytoplasm in which this enzyme (LDH) exists. We used an LDH Cytotoxicity Detection Kit (Roche). LDH activity was measured in culture supernatants using F Mulsiskan FC (Thermo Scientific) at λ = 490 nm with a λ = 600 nm wavelength reference. Triton-X was used as a control for cell damage.

The experiments were performed in triplicate. The significance values were calculated using the T-test and the Graphpad Prism software. Cell morphology was evaluated using an inverted Olympus IX73 microscope after 24 h of incubation with the materials.

## 3. Results and Discussion

### 3.1. EDXRD Measurements

**Figure 1:**
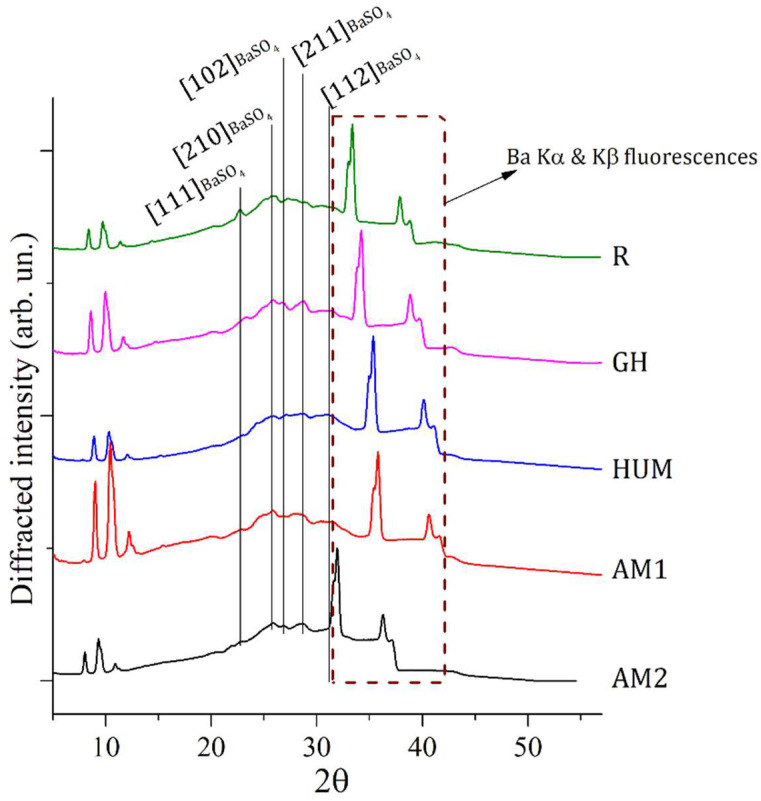
EDXRD spectra collected on 5 different composite samples made of PMMA-BaSO_4_-BPO as major components. Most intense Bragg reflections, all belonging to the PMMA component, have been labeled with the proper Miller indexes. Fluorescence contributions of Ba are visible in correspondence of the region within 30 to 40°.

EDXRD spectra collected on samples R, AM1, AM2, HUM, and GH as diffracted intensity as a function of scattering angle 2θ are plotted in Figure 1. Spectra collected in Energy Dispersive mode are registered in a spectroscopic fashion (i.e., as a function of energy). Afterward, ED diffraction patterns were converted into angle-dependent ones by using the formula:

(1) $$2\theta =2\sin^{-1}\left(\frac{q}{a\cdot E}\right)$$

where q is the scattering parameter, E is the energy of the diffracted photon, and a is a constant (1.014 Å^−1^/KeV).

All the diffraction patterns in Figure 1 are characterized by broad and convoluted signals indicative of an almost amorphous status of the samples. Deconvolution of signal within the region of interest (ROI) between 20 and 30°, where the majority of diffraction signals are located, allowed to individuate peak positions of specific Bragg reflections, which contribute to the selected ROI. Attributions of EDXRD deconvoluted peaks representative of all samples (R, GH, HUM, AM1, AM2) are reported in Table 2.

The region within an angular range of 30 to 40° is characterized by the presence of a fluorescence signal of Barium present in all samples as an elemental constituent of barite (BaSO_4_) [PCPDF #09-0169]. EDXRD source is constituted by polychromatic radiation ranging from 0 to 50 KeV and, therefore, able to excite fluorescence with characteristic energy contained in this range, as the ones of Ba: Ka (32 KeV) and Ba: Kb (36 KeV).

### 3.2. Raman Measurements

**Figure 2:**
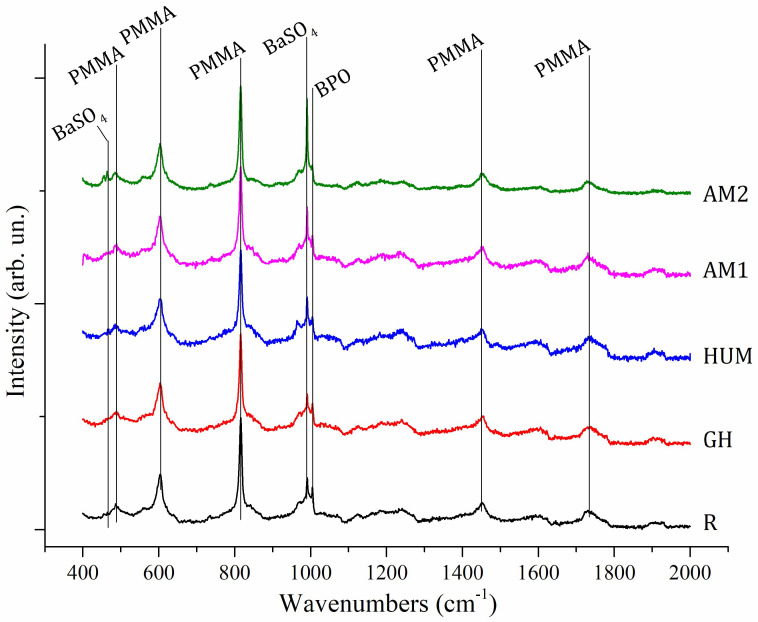
Raman patterns collected from 5 different composite samples made of PMMA−BaSO_4_ −BPO as major components. Most intense bands have been labeled and assigned to their respective component of the composite.

Figure 2 shows Raman spectra collected on samples R, AM1, AM2, HUM, and GH. The plotted spectra share many similarities due to the similar composition of the different samples, which are prepared with the same solid components (PMMA, BaSO_4_, BPO). All patterns were collected with a red laser source (785 nm). Second-order polynomial baseline correction was applied as a post-acquisition process to remove fluorescence contribution.

Attribution of Raman shifts related to most intense bands was attempted by comparison with literature data [47,48,49] and reported in Table 3.

By comparison, only minor differences are detectable within spectra and consist of differences in relative intensities of specific bands. The most relevant samples are AM1 and AM2, with relative higher signals of shifts at 990 cm^−1^ attributed to ν_1_(SO_4_) of BaSO_4_, and the sample R with a higher intensity associated with the shift assigned to C_6_H_6_ breathing at 1000 cm^−1^ of BPO component.

Some correlations between bulk and surface-related properties of the samples can be attempted by comparison of results between EDXRD and Raman investigations. Indeed, while X-ray-based investigation techniques (e.g., EDRXD) can deeply penetrate into the samples and, therefore, are able to return information from the bulk, Raman spectroscopy, in reflective mode, can investigate only the surface of the specimen with a penetration depth of a few microns. All EDXRD samples show signals related to barite, suggesting that this phase is present in the bulk of all samples. On the other hand, only AM1 and AM2 Raman spectra return the presence of stronger barite Raman signals. This experimental evidence suggests that among all specimens, surfaces of samples AM1 and AM2 are richer in barite phase. These outcomes could be interpreted as a better distribution of this component throughout the sample bulk/surface. More homogeneous distribution of barite can be correlated with improved mechanical properties, such as the elastic modulus in flexure and the flexural strength. This is coherent with the experimental evidence obtained from the mechanical tests carried out in this work and also in good agreement with the literature results, as barite is a well-known additive for polymers-based composites, able to improve mechanical properties and, in particular, tensile strength and hardness if homogeneously distributed within the composite [50,51].

### 3.3. Thermogravimetric Analyses

**Figure 3:**
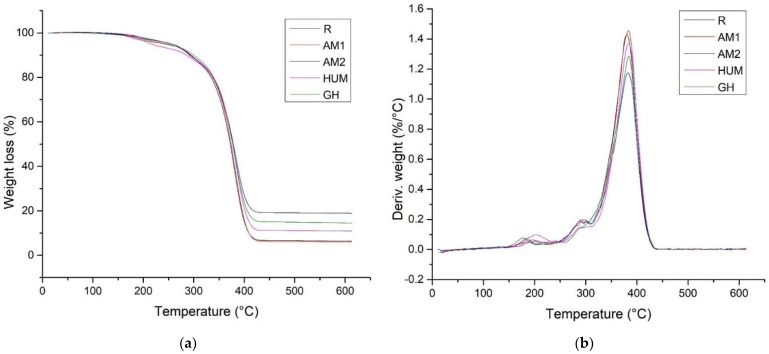
TGA (**a**) and DTG (**b**) curves of reference R and experimental bone cement.

We studied the thermal stability of all bone cement through thermogravimetric analysis. The weight loss curves (TGA) and derived weight curves (DTG) for experimental bone cement compared to the reference are shown in Figure 3.

In Figure 3, it can be noticed that the TGA curves for all the investigated samples were leveled at temperatures higher than 430 °C, which indicates a low weight loss beyond this temperature. Table 4 presents the TGA analysis for the investigated bone cement.

We tested the thermal stability of the bone cement by measuring the decomposition temperature at 10% mass loss (T_10_). There is an improvement in the thermal stability of the cement by adding antimicrobial agents, except in the case of the HUM sample. Adding peppermint essential oil incorporated in hydroxyapatite, the T_10_ was shifted to lower values, which is caused by the loss of volatile compounds in the oil.

As it is well-known, the inflection temperature is the peak point of the DTG curve, and a higher value of this temperature indicates high thermal stability. As seen from the DTG curves and Table 4, the presence of antimicrobial agents induces a slight increase in this temperature. We can conclude that the addition of antimicrobial agents had no significant influence on the thermal stability of the experimental bone cement.

### 3.4. Mechanical Properties

The main function of bone cement is to transfer the load from the prosthesis to the bone; thus, its effectiveness is viewed through its mechanical properties [52,53].

**Figure 4:**
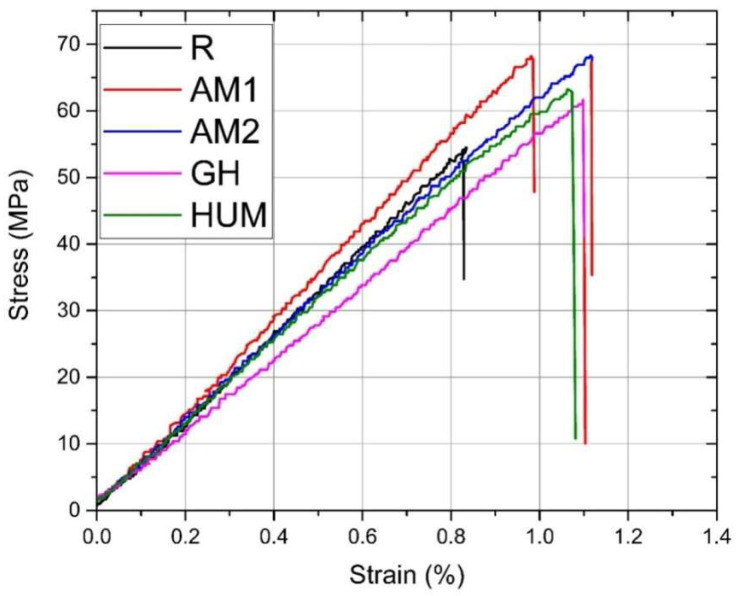
Selection of representative stress–strain curves in flexure.

*Flexure tests.* A selection of representative stress–strain curves in flexure is depicted in Figure 4, which shows a brittle behavior in this loading type. All samples, regardless of formulation, failed almost immediately when the stress exceeded the elastic limit without any significant signs of plastic deformation.

**Figure 5:**
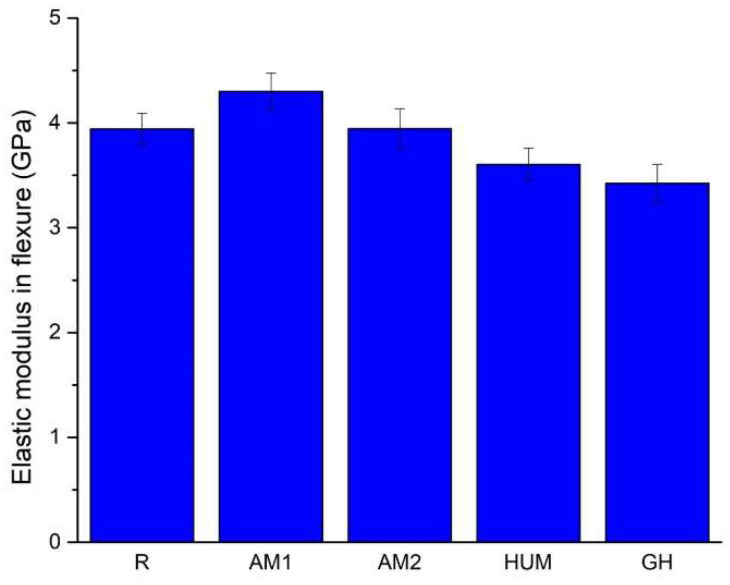
The average value of the elastic modulus in flexure for the tested samples.

A bar chart comparing the elastic moduli is presented in Figure 5, which is in accordance with the experimental data presented in Table 5. It can be seen that the mean values range from 3.42 GPa (GH sample) to 4.30 GPa (AM1 sample).

Adding gentamicin (GH samples) and hydroxyapatite with incorporated peppermint essential oils (HUM samples) leads to a decrease by 13% and 9% of the mean value of the elastic modulus compared to the reference sample R.

The addition of silver nanoparticles incorporated in the ceramic glass shows a 9% increase for AM1 samples and less than 1% for AM2 samples compared to the reference sample R. In this current research stage, an improvement of the elastic modulus can be achieved up to 2% silver particles addition; beyond this infill, a decrease can be inferred.

According to International Standard ISO 5833 [54], the minimum value of the modulus of elasticity in flexure, when tested in a three-point bending configuration, needs to be at least 1.8 GPa, a criterion that all formulations have met.

**Figure 6:**
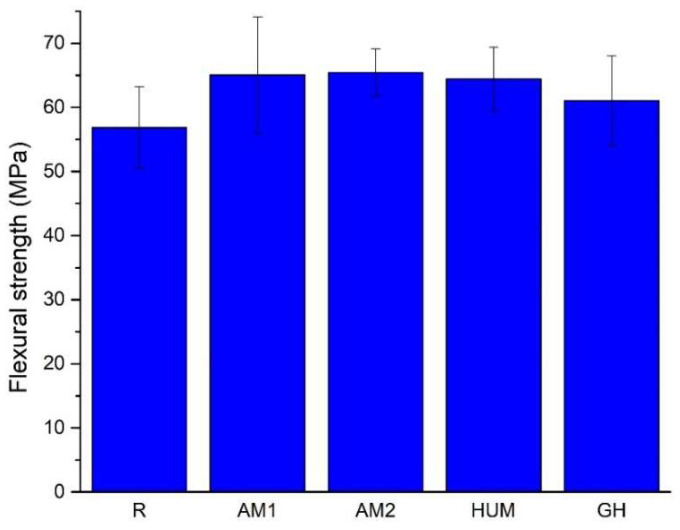
The average value of the flexural strength for the tested samples.

The average values of the flexural strength, presented in Figure 6, reveal that adding antimicrobial agents to the formulation yields an increase of 3.7% for the GH samples, 9.5% for HUM samples, 10.5%, and 11.4% for AM1 and AM2 samples, compared to the reference sample R. This increase most likely can be attributed to the change in load distribution in the tensile region of the specimens.

Adding antimicrobial additives does not strongly influence the obtained flexural strength results, but a slight change can be attributed to the addition of a carrier for antimicrobial agents, as the ANOVA test results reveal. All recorded values are above the limit required by International Standard ISO 5833, at least 50 MPa.

Given the limited information available within this research, a statistical analysis was performed using one-way ANOVA on the values obtained for the elastic modulus and flexure strength. At α = 0.05, the ANOVA test results showed that the addition of antimicrobial agents influences the bone cements mechanical characteristics and that by using the previously presented amounts, the required mechanical requirements for safe use are met.

**Figure 7:**
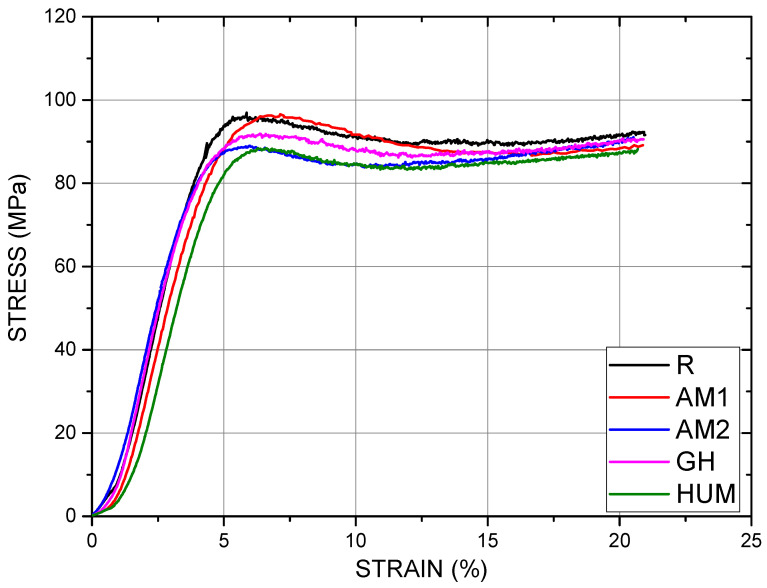
Selection of stress-strain curves in compression from the tested samples.

*Compression test.*Figure 7 shows a selection of stress-strain curves in compression to depict the mechanical behavior of the tested bone cement. The curves reflect a malleable, deforming plastically by shortening and barreling. Given this behavior, similar to a malleable metallic material, using the maximum strength as a descriptor is inappropriate, so as a consequence, the strength at 0.2% strain is applied. The procedure involved to determine this parameter is the same as for the yield strength in tension, but when used in compression, a prior noise compensation is required.

Regardless of the addition of the antimicrobial agent, the mechanical behavior was primarily similar in the elastic region.

**Figure 8:**
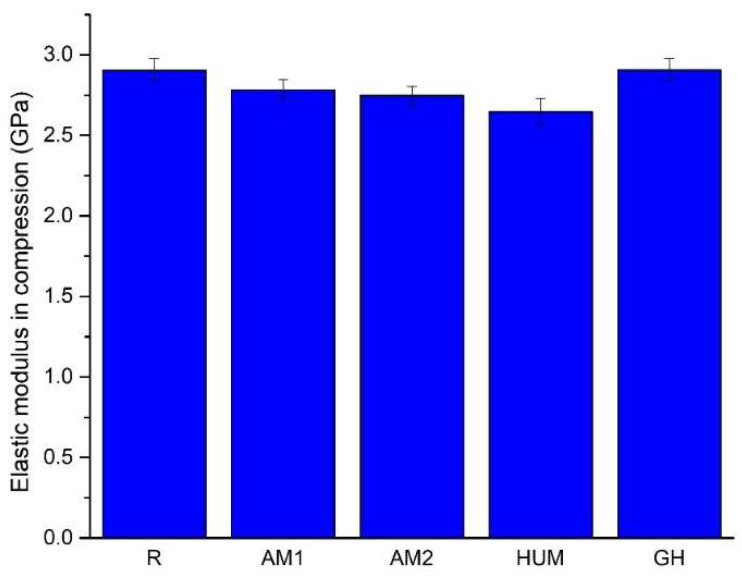
The average value of the elastic modulus in compression.

A comparison of the average value of the elastic moduli of the tested bone cement is presented in Figure 8 and in Table 6.

The addition of gentamicin (GH samples) does not influence the elastic modulus of the bone cement, while the addition of silver nanoparticles incorporated in ceramic glass (AM1 and AM2 samples) leads to a slight decrease, roughly 4% and 5% of its values compared to the reference sample R. The addition of peppermint oil in hydroxyapatite shows a more substantial decrease, by roughly 9% reported in the sample R.

**Figure 9:**
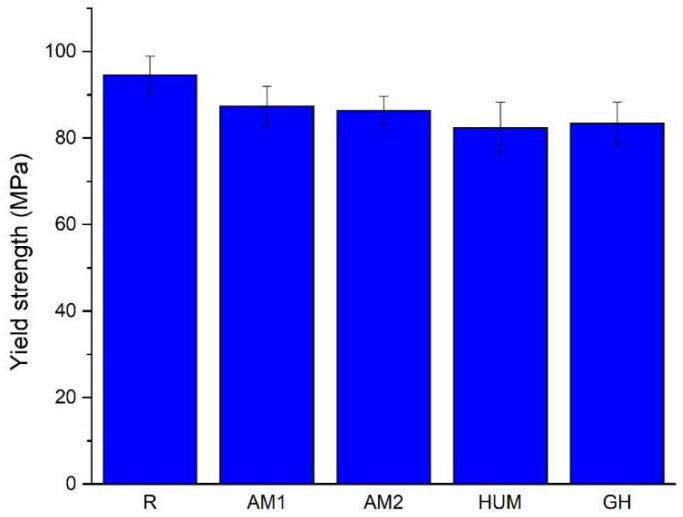
The average value of the yield strength.

A comparison of the average yield strength values is presented in Figure 9, which shows that adding an agent leads to a decrease. Most likely, this agent acts like a discontinuity within the material rather than a reinforcing agent.

With the addition of silver nanoparticles incorporated in ceramic glass (AM1 and AM2 samples), the yield strength drops by roughly 8% and 9%, while with the addition of gentamicin (GH samples) and peppermint oil in hydroxyapatite (HUM samples), a higher drop of 11% and 12% is observed. All reports are compared to the reference sample R.

Again, one-way ANOVA is used to verify that the addition of antimicrobial agents can be considered an influencing factor on the mechanical properties (i.e., the elastic modulus and yield strength) of the bone cements. At α = 0.05, the ANOVA results suggest that there is a statistically significant difference, and that the antimicrobial agent addition can be considered an influencing factor.

Despite that in pure compression, the addition of antimicrobial agents leads to a decrease in mechanical characteristics, this decrease is of an acceptable amplitude (maximum 12%, compared to the reference sample R), and the strength values for the investigated bone cement still meet the minimum requirements stipulated by ASTM F451 standard (i.e., the strength of at least 70 MPa) [55].

### 3.5. Biocompatibility Properties

**Figure 10:**
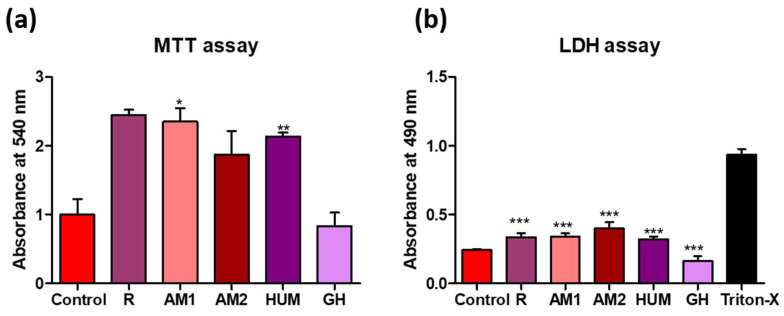
Biocompatibility analysis of tested bone cement. (**a**) Viability and proliferation potential of the samples (Statistical significance: * *p* < 0.05; ** *p* < 0.005). (**b**) Cytotoxic response of the G292 osteoblasts in the presence of the samples (Statistical significance: *** *p* < 0.001); *n* = 3.

**Figure 11:**
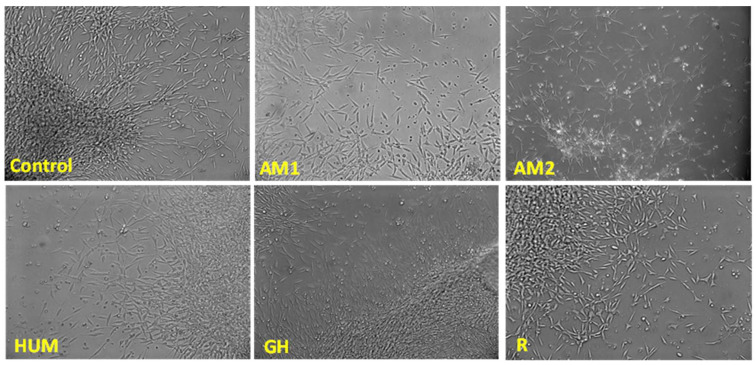
Cell morphology in the presence of the investigated samples, phase-contrast microscopy, 10× magnification.

Among the tested bone cement, AM1, HUM, and R exhibited the highest degree of biocompatibility, as revealed by the MTT and LDH tests (Figure 10). Samples GH showed the lowest cytotoxicity (as demonstrated by the low LDH values), yet it did not stimulate cell proliferation as much as the other materials tested. None of the materials tested induced significant changes in the cell morphology, as shown in Figure 11.

## 4. Conclusions

In this study, four PMMA bone cements modified with different antimicrobial agents were obtained as potential biomaterials to be applied in orthopedics for endoprosthesis fixation. It has been shown that the addition of antimicrobial agents improves the flexural strength of the traditional PMMA bone cement, while the yield strength values show a decrease, most likely because this agent acts as a discontinuity inside the material rather than as a reinforcing agent.

All tested bone cements seem to have three stages of degradation, the third peak being the main one (at 380–385 °C). This peak, due to the polymeric chains’ scission, is shifted to a higher temperature for AM1, AM2, HUM, and GH samples compared to the reference sample R. In the case of all investigated samples, the addition of antimicrobial agents had no significant influence on the thermal stability.

The bone cements showed good biocompatibility and the possibility of osteoblast proliferation (MTT test) along with a low level of cytotoxicity (LDH test).

Our previous results [1] have demonstrated that the incorporation of peppermint essential oil incorporated in hydroxyapatite, gentamicin, and silver nanoparticles incorporated in ceramic glass (4%) (HUM, GM, and AM2 samples) generated clear areas of inhibition against *Staphylococcus aureus* ATCC 25923 and *Pseudomonas aeruginosa* ATCC 27853.

Our research indicates that bone cements with peppermint essential oil incorporated in hydroxyapatite and silver nanoparticles incorporated in ceramic glass used as antimicrobial agents can be a viable solution that can be introduced in the near future in common clinical practice.

## Figures and Tables

**Table 1:** Composition of the new antimicrobial bone cements.

Samples	Components	Antimicrobial Additive
R	Powder 40 g (87.6% PMMA, 2.4% BPO, 10% BaSO_4_) + Liquid 14.4 g (85.3% MMA, 13.2% BMA, 1.5% DmpT, 20 ppm HQ)	None
GM		5% gentamicin—refers to the total powder weight
HUM		5% peppermint essential oil incorporated in hydroxyapatite (HAp)—refers to the total powder weight
AM1		2% silver nanoparticles incorporated in a ceramic glass—refers to the total powder weight
AM2		4% silver nanoparticles incorporated in a ceramic glass—refers to the total powder weight

Methyl methacrylate—MMA; Butyl methacrylate—BMA; *N*,*N*-Dimethyl-p-toluidine—DmpT; Hydroquinone—HQ; Poly(methyl-methacrylate)—PMMA, Benzoyl peroxide—BPO, Barium sulfate—BaSO_4_; Hydroxyapatite—HAp.

**Table 2:** EDXRD deconvoluted peaks of samples R, GH, HUM, AM1, AM2 [46].

Peak Position (°)	Assignment	Relative Intensity	Component
22.8	[111]	52%	BaSO_4_
25.8	[210]	100%	BaSO_4_
26.9	[102]	70%	BaSO_4_
28.7	[211]	99%	BaSO_4_
31.5	[112]	50%	BaSO_4_

**Table 3:** Attribution of Raman shifts identified in experimental bone cements.

Raman Shift (cm^−1^)	Assignment	Component
463	M–O_12_	BaSO_4_
487	Out of plane deformation	PMMA
603	Deformation O–C=O	PMMA
812	Symmetric stretching *v_s_*(C–O–C)	PMMA
990	ν_1_ (SO_4_)	BaSO_4_
1000	C_6_H_6_ breathing	BPO
1450	CH_2_ Deformation	PMMA
1730	Stretching C=O	PMMA

**Table 4:** TGA analysis of investigated bone cement.

Sample	Inflection Temperature at the Highest Rate of Weight Loss (°C)	Mass Loss at Inflection Temperature (%)	T_10_ (°C)	T_50_ (°C)	Residue Content at 600 °C (%)
R	380.03	39.17	292.83	372.28	6.41
AM1	383.14	36.91	291.65	373.86	6.0
AM2	382.68	44.83	294.51	378.12	18.92
HUM	383.71	40.92	285.53	376.96	10.93
GH	384.41	40.86	299.46	377.02	14.54

**Table 5:** The elastic modulus in flexure and flexural strength results of the experimental bone cement.

Samples	Elastic Modulus in Flexure [GPa]	Flexural Strength [MPa]
R	3.94 ± 0.15	56.83 ± 6.36
AM1	4.30 ± 0.17	65.03 ± 9.09
AM2	3.94 ± 0.18	65.46 ± 3.69
HUM	3.60 ± 0.15	64.44 ± 4.92
GH	3.42 ± 0.67	61.01 ± 7.04

**Table 6:** The elastic modulus in compression and yield strength results of the experimental bone cement.

Samples	Elastic Modulus in Compression [GPa]	Yield Strength [MPa]
R	2.90 ± 0.07	94.60 ± 4.34
AM1	2.78 ± 0.06	87.40 ± 4.56
AM2	2.74 ± 0.05	86.40 ± 3.29
HUM	2.64 ± 0.08	84.40 ± 5.86
GH	2.90 ± 0.06	83.40 ± 4.83

## Data Availability

The experimental data on the results reported in this manuscript are available upon official request to the corresponding authors.
